# Dolichocolon Presenting with Bilateral Hydronephrosis in a Neonate

**DOI:** 10.1155/2021/6658525

**Published:** 2021-03-19

**Authors:** Laura Gielen, Anke Raaijmakers, Bert De Groote, Eva ter Haar

**Affiliations:** ^1^University of Antwerp, Department of Pediatrics, Antwerp, Belgium; ^2^Department of Pediatrics, Hospitals ZNA Jan Palfijn, Merksem, Belgium; ^3^University of Leuven, Department of Pediatrics, Leuven, Belgium

## Abstract

**Background:**

Dolichocolon is an inborn anatomic variant of the colon with redundancies often causing constipation and/or volvulus presenting in childhood, adolescence, or adulthood. To the best of our knowledge, this is the first case of dolichocolon presenting in infancy with constipation and bilateral hydronephrosis. *Case Presentation*. A nineteen-day-old neonate presented to the emergency department with severe constipation and discomfort. During his admission, he developed pyelonephritis, and subsequent ultrasound of the kidneys and bladder showed bilateral hydroureteronephrosis. A barium enema was performed and it showed a dolichocolon. Enemas and lactulose were initiated with good effect on both the constipation as well as the hydronephrosis.

**Conclusions:**

Dolichocolon in a neonate can cause severe constipation which could also lead to an obstructive nephropathy if untreated. Monitoring of urine flow might be indicated when a neonate presents with severe constipation.

## 1. Introduction

Dolichocolon is an inborn anatomic variant of the colon, where redundancies may be located in the right, middle, and left part of the colon and at the flexures [1]. In a study by Brumer et al. [2] in which 53 patients had a barium enema for reasons other than constipation, only one patient (1,9%) had a redundant colon; on the other hand, Larimore and colleagues [3] found redundancies in 28.5% of 562 cases of patients of all ages with constipation who underwent a barium enema. They found the same frequency and variation in neonates as in adults [1]. The exact prevalence remains unknown. The most common presenting symptoms of dolichocolon include constipation, abdominal pain, and volvulus [1].

The link between constipation and urinary tract problems in children, including infections, enuresis, vesicoureteral reflux, and upper renal tract dilatation is known and has been described by Averbeck and colleagues [4]. They concluded that the underlying pathophysiology of these findings has not yet been clearly defined [4]. Here, we report a case of a nineteen-day-old neonate with dolichocolon and severe constipation presenting with bilateral hydroureteronephrosis.

## 2. Case Presentation

A nineteen-day-old neonate presented to the emergency department with generalized discomfort, decreased oral intake, and increased abdominal distension. Clinical examination showed an irritable neonate with a tender abdomen and a marbled skin. Abdominal X-ray showed fecal impaction, but there were no signs of pathological bowel dilations or signs of strictures. Because of the discomfort and young age of this neonate, he was admitted to the Department of Pediatrics for further observation with the clinical suspicion of severe constipation.

From his medical history, we noted that he was born at term and there were no perinatal problems. He passed meconium within 12 hours after birth. One week prior to hospital admission, he presented to the outpatient clinic with difficult defecation and a little perianal fissure, which improved spontaneously after three days. There was no family history of bowel problems.

During his admission, he developed fever (38.5°C), and subsequent blood analysis showed a CRP of 92.8 mg/L (ref. <5 mg/L) without leukocytosis (8.81 10E9/L; ref. 5.0–19.5 10E9/L). Other laboratory findings were normal (hemoglobin 12.8 g/dL (ref. 10.0–18.0 g/dL), thrombocytes 382 10E9/L (ref. 253–493 10E9/L), urea slightly decreased 3.5 mg/dL (ref. 11–36 mg/dL), and creatinine 0.23 mg/dL (ref. 0.31–0.88 mg/dL)). Thyroid function was normal (free T4 20.3 pmol/L (ref. 11.5–28.3 pmol/L) and TSH 1.55 mU/L (ref. 0.72–11.00 mU/L)). Urine analysis showed 13728 WBC/*µ*L (ref. <15/*µ*L) and 35 erythrocytes/*µ*L (ref. <19/*µ*L). Because of the suspicion of pyelonephritis, empirical antibiotics (third-generation cephalosporin) were started after blood collection and clean catch urine collection. Later, urine culture showed >100 000 CFU/ml *E. coli*. Blood cultures remained sterile.

**Figure 1:**
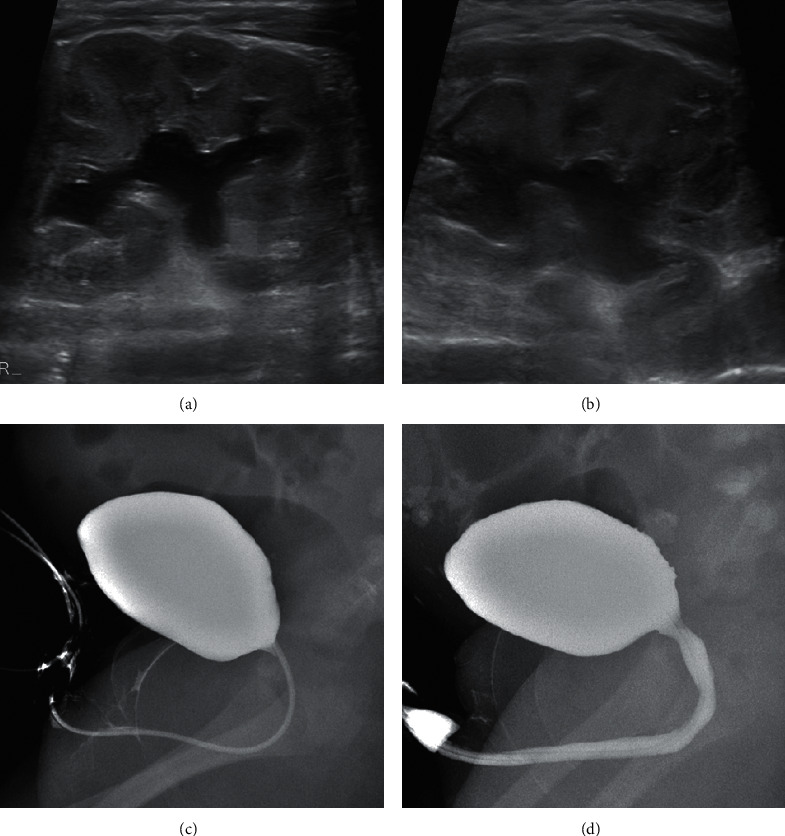
Renal and bladder imaging. (a). Hydronephrosis of the left kidney. (b). Hydronephrosis of the right kidney. (c). Micturation cystourethrogram (filling phase-normal bladder wall). (d) Micturation cystourethrogram (voiding phase-normal urethra).

**Figure 2:**
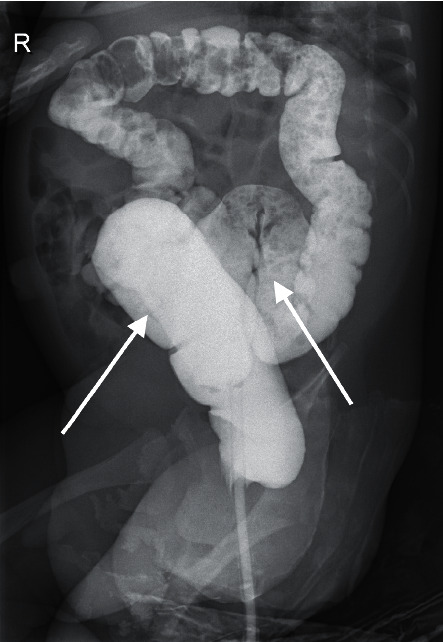
Barium enema X-ray. Dolichocolon (additional curves at sigmoid and transverse colon, arrows), no strictures, no pathological bowel dilatations, and no signs of Hirschsprung's disease.

Ultrasound of the kidneys and bladder showed a bilateral hydronephrosis (Figures 1(a) and 1(b)). The diameter of left pyelum was 12 mm, the diameter of right pyelum was 17 mm, and both the left and the right proximal ureter were visible, 8 mm and 11 mm, respectively (we considered values of pyelum <10 mm and invisible ureters as normal). There was the suspicion of an obstruction on the level of the vesicourethral junction bilaterally which could not be confirmed on MAG3 scan. A micturition cystourethrogram was normal (Figures 1(c) and 1(d)) without a significant residue (<1 ml). Bladder anatomy was considered normal despite suboptimal filling. A (‘hot') DMSA-scan was performed to rule out infection and/or cortical defects and was reported normal. Because of the increased abdominal distension and difficult defecation, a barium enema was performed and it showed a dolichocolon with redundancies at the level of the sigmoid and the transverse colon (Figure 2). Radiology images were not suggestive for Hirschsprung's disease (Figure 2) which was also ruled out by rectal biopsy.

Further investigations (sweat test, to exclude constipation in context of cystic fibrosis, ultrasound of the sacrum, and transfontanellar ultrasound) were normal. Five days after starting antibiotics, a control urine sample was sterile and oral antibiotics (ciprofloxacin) were continued for a total of 10 days. Subsequently, urinary prophylaxis was initiated. Micturition cystography was normal with no residual volume after micturition (<1 ml).

During the hospitalization, there was almost no spontaneous defecation, so oral lactulose (Duphalac®) and enemas with physiologic solution (20 ml/kg three times a day) were started in consultation with a gastroenterologist. This led to major clinical improvement and resolution of symptoms. After treating the constipation, the ultrasound of the kidneys showed a remarkable reduction of the hydronephrosis (left pyelum diameter 8 mm, right pyelum diameter 5 mm) which completely resolved at the subsequent scan. Urinary prophylaxis was ceased after 6 months, and until now, there was no recidivism of urinary tract injection. At present, there are no developmental concerns and the constipation has not reoccurred. Follow-up will be continued by our general pediatric team.

## 3. Discussion and Conclusions

To the best of our knowledge, this is the first case describing dolichocolon in a neonate presenting with bilateral hydroureteronephrosis. Treating the constipation led to a resolution of both renal and bowel symptoms.

The systematic review of Averbeck described the link between constipation and LUTS [4], but the underlying pathophysiology remains unclear. Based on an extensive literature review with PubMed search terms “constipation” and “hydronephrosis OR obstructive nephropathy,” we found 6 cases of children with constipation presenting as an obstructive nephropathy (Table 1). The youngest case with constipation and an obstructive nephropathy was an almost two-year-old toddler with a history of Hirschsprung's disease [10]. In all cases, there was a history of severe constipation for several months or years. In contrast, our case presented in infancy with a history of constipation for two weeks. In some of the previously described cases, there were also signs of kidney failure. However, our case presented early, and treatment was initiated soon after presentation without any renal compromise. Treatment was comparable in all cases: treating the constipation cured the obstructive nephropathy as we also saw in our case. In two cases, there was a surgical intervention needed to remove the fecaliths [5, 6], and in one case, even a partial colectomy was needed [6]. In some cases, the use of a transurethral catheter was needed to evacuate the urine from the bladder [6]; however, urine outflow was never compromised in our case.

The study of Akl [11] described occult chronic functional constipation as a cause of reversible hydronephrosis. In this study, there was always a history of chronic constipation unlike in our case. Similarly, the hydronephrosis resolved in all nine cases after treatment with oral lactulose [11].

In conclusion, dolichocolon in a neonate can cause severe constipation which could also lead to an obstructive nephropathy if untreated. Monitoring of urine flow might be indicated when a neonate presents with severe constipation.

## Figures and Tables

**Table 1:** Constipation presenting with hydronephrosis.

Characteristics	Patient 1	Patient 2	Patient 3	Patient 4	Patient 5	Patient 6
Sex	Male	Male	Male	Female	Male	Female
Age (years)	2 y	17 y	12 y	8 y	10 y	2 y
Clinical symptoms	No bowel motions, lethargy, irritability, oliguria	Generalized fatigue, anorexia, abdominal distension, oliguria, bilateral lower limb edema	Chronic abdominal pain, anorexia	Unknown	Chronic constipation, soiling abdominal bloating, nausea, anorexia	Abdominal pain, agitation, abdominal distention, voiding difficulty
Medical history	Prematurity (34 weeks of gestation), chronic constipation, global developmental delay	Chronic constipation with overflow incontinence	Severe autism	Frequent, nonfebrile, urinary tract infections, severe constipation	Chronic constipation	Hirschsprung's disease (Duhamel operation), chronic constipation
Obstructive nephropathy	Bladder distention, renal failure (creatinine 140 *µ*mol/L)	Bilateral hydroureteronephrosis, distended and compressed urinary bladder, renal failure (creatinine 72 *µ*mol/L)	Right hydroureteronephrosis	High-grade obstruction of the right upper pole and left lower pole moieties	Compression of the bladder, dilatation of left pelvicalyceal system and left ureter	Acute urinary bladder neck obstruction, bilateral hydronephrosis
Therapy	Transurethral catheter, surgical review, manual evacuation of the rectum	Transurethral catheter, diuretics, large bowel resection (∼1 m)	Not known	Aggressive bowel preparation	Aggressive macrogol therapy and fleet enema	Manual evacuation, laxatives, reoperation of Duhamel procedure
Reference	[5]	[6]	[7]	[8]	[9]	[10]

## Data Availability

All data generated or analyzed during this study are included within this article.
